# Dental Wreath—Original Poem

**Published:** 1856-12

**Authors:** 


### DENTAL REGISTER OF THE WEST.

VOL. X.—DECEMBER, 1856.—NO. 2.

### Original Essays and Communications.

### DENTAL WREATH.

The attractive flowers that compose this beautiful wreath of poesy were selected from the unpoetical fields of dentistry by a friend of my early youth, who did not intend it for public gaze and criticism, but reluctantly consented, on condition she should remain incognito.

The knowledge of the subject treated, and the merits of the poem as a literary effort, reflect much credit on the authoress. Public opinion has already conceded to dentistry an important position among the professions. Its growing usefulness in relieving the pains and ills of man, the increasing number of colleges for qualifying young men for the responsibilities of the profession, the social position of the gentlemen embarking in it, the character and number of the journals sustained by those in practice—all warrant us in the belief that dentistry, particularly in the United States, is assuming a rank among the first of the professions. The desire to contribute a mite to the literature of our young science, and to encourage the commendable literary taste of professional brethren, induces me to offer this little production for publication. If *any* derive pleasure from its perusal, the slight trouble I have taken in bringing it before them, will be richly rewarded; and to *those* I beg leave to say, I sincerely regret the modesty of our "*gifted child of song*" prevents my informing them to whom they are indebted. As it is, I humbly submit it to the dental profession, hoping this will be a satisfactory apology for my offering, and for its anonymous appearance. CHS. H. ROBERTS.

*Poughkeepsie, N. Y., Oct. 24th,* 1856.

TO DRS. C. H. AND W. B. ROBERTS,

WHO HAVE FOUND TIME, AMID THE CARES OF BUSINESS AND THE EXCITEMENTS OF THE WORLD, TO KEEP ALIVE THE FRIENDSHIPS OF EARLY YEARS,

THIS POEM IS MOST RESPECTFULLY INSCRIBED.

### CANTO FIRST.

### THE ARGUMENT.

Supremacy of the light of Genius shown, over the great lights of the material universe. The sublime and invincible character of the soul in which it reigns. Objections answered. Desolations of the intellectual world, were genius blotted from existence. What it has done to benefit mankind, when directed by philanthropy. Dentistry proposed, in connection with other blessings conferred by intellectual and artistic power, suggested and controlled by genius. Its influence upon health, and hence upon happiness.

The light of stars is pure and bright,
Soft glowing in the heaven of night;
The moonlight has a holy gleam
As fairy as a spirit's dream;
Each sunbeam is a joyous thing,
With blessings on its golden wing;
And yet these glorious beams divine
But faint, and few, and feebly shine
Beside the light of that high soul
Where genius holds supreme control.
It loves to seek the works of Him
Before whose eye the stars wax dim,
Rejoicing in his matchless might,
Who dwells in uncreated light.
It searcheth far, and soareth long,
Where circling spheres move on in song,
Inspiring minds o'er boundless space
Their laws and motions all to trace.
Though storm-clouds, charged with ruin, lower
O'er him who hath this godlike power,
Unfaltering soars he still.
  The lightning's flash, the thunder's crash,
Move not his sovereign will.
And when from out yon azure sky
He earthward bends his eagle eye,
All forms of matter, at his word,
  Their treasures wide display,
And mysteries before unheard
  Lie open to the day.
The mould that lies beneath his tread,
The meteor sailing o'er his head,
The crystal drops, so pure and sweet,
That flow in music at his feet,
The loftiest tree, the humblest flower,
In forest dell, or garden bower,
With beauty and with meaning fraught,
Have furnished scope for deepest thought.
Beyond the outer form he sees,
And traces strange affinities,—
Where others look with vacant eye,
To him are wonders deep and high.

But where's the good? the objector cries;
May not the lowly, too, be wise?
Is mind the purer, earth the brighter,
Home the happier, hearts the lighter,
For all these minds of might?
Go, in thine insolence inquire
Of Him, who placed yon orb of fire
To bless the world with light,
Why he did form that dazzling thing,
To scorch us with its fiery wing?
Quench thou the sun, then lift thine eye,
In wonder to the evening sky,
And ask for Dian's silver ray,—
Still the starbeams sparkle bright,
But will their million threads of light,
When torn the day-god from his throne,
Awake the buds that slumber lone
Through winter's chill and dreamless day?
Or on the lakelet's waters blue,
The lily's shining robes unfold,
Or sweetly tinge its virgin hue
With that light fringe of liquid gold?
Go, quench the intellectual sun,
Ere half its glorious race is run,
The twinkling of the stellar host,
Methinks would scarce remain,
Bewildered should we cry, and lost,
Oh! light that radiant orb again!

All that minds by genius taught,
The wonders they on earth have wrought,
And all the've suffered, felt, and thought,
Attempt how vain to say!
The stake, the block, the dungeon cell,
Can of their fate, their glory tell;—
But these stern times have passed away,
And rises fair a brighter day.
The world applauds her sages now,
And wreathes a laurel on each brow.
Her wise men who have deeply felt
How meager human lore,
Who at science' shrine have knelt,
And dauntless sought for more,—
And then with philanthropic heart,
Have turned all science, knowledge, art,
From vague abstractions, dark and wild,
Where sage and alchymist of yore
Had conned their spells and cycles o'er,
To where the light supreme hath smiled—
*These* are the names that brightest shine
With radiance pure, almost divine.

What such as these have thought and done,
What triumphs they have nobly won,
Is registered by deeds sublime,
That cannot die with things of time,

It were not ours to swell the strain,
For sure such effort would be vain;
And yet we see along our way
Their works with every passing day;
We see light poured o'er darkened lands,
And bound the earth in kindred bands,
Till man, a stranger now no more,
Shall brothers find on every shore;
We see the steam-king, in his might,
Outstripping e'en the sea-bird's flight,
To breast in scorn the mountain wave,
And bid the tempest be his slave.
We see for thought electric wires,
Where all its wild, ethereal fires
With lightning flash speed on their course
Swifter than day-beams from their source.

Though th' elements man's voice have heard,
And bowed obedient to his word,
A better knowledge he has gained,—
Has learned to live as Heaven ordained.
The mechanism of his frame,
   So nicely planned and delicate,
Its substance changing, yet the same,
   So simple, yet so complicate,
Has been unfolded to the light,
And wondrous things his search revealed,
That had been long, too long concealed—
   Lost in shades of gloomy night.
Though some have deemed themselves too wise
To show such truths to other eyes,
*A few unselfish* have been found,
Who have this prejudice unbound—
Who have stood forth and boldly taught
That all the ills with life inwrought
Proceed from violated laws,—
And this the sole, the certain cause
Of early death, disease and pain;—
Let not such warning pass in vain!
The laws that govern every part,
From simplest hair to throbbing heart,
The constant care that each should take,
Lest he these laws of nature break,
Have been set forth—hence all may know
What blessings knowledge can bestow;
And each with length of days be blest,
Ere going to his final rest.

In close connection here we bring
A topic new for bard to sing.
A science known to classic fame,
Though scarce it had an honored name,
Till *Genius* raised it from abuse,
And labored to exalt its use,
To banish ignorant pretense,
And hedge it round with a defense

Of knowledge that must ever be
A bright, enduring panoply.
Though unappreciated long,
Its friends did suffer many a wrong;
Their high achievements hence must live
Blessings numberless to give.
Its vast importance we would tell,
In numbers of such potent spell,
That they should speak to every ear
In tones convincing, loud, and clear,
That all may know, and knowing, feel
That health and beauty for their weal
Upon its teachings must depend—
To all a blessing, and a friend.

Devoid of fancy's airy wings,
Stern facts *may seem* presuming things
To don the golden robes, so bright,
Of Poesy, that child of light;
And yet they *may* not be unmeet
For fair array, and numbers sweet;
Though strangers most to rules of song,
To Truth's fair province they belong;
Therefore pause we not to dream,
While Dental Science is our theme.
And first we claim that rosy health,
Dearer than India's princely wealth,
Without its influence and its aid,
Has oft been doomed to droop and fade.
One means it is, but known aright,
Can put a thousand ills to flight,
And unimpaired this treasure keep,
O'er loss of which pale thousands weep.
The tiny architects that fashion
Each portion of our frame,
That take the atoms of nutrition,
To feed the vital flame,
Can not their office well perform,
And strength and vigor must decay,
And life be but a winter's day,
Obscured by gathering cloud and storm,
Unless the teeth with strictest care
Shall all the aliment prepare;—
One link they form in that great chain
Which serves existence to sustain;
Frustrated once fair nature's plan,
Diseases in a hideous clan,
With stealthy step come creeping on,
Till health, and joy, and life are gone.
Dyspepsia one, that hydra thing,
That does such horrors with it bring,
And every hapless nerve unstring.
Its thousand nameless ills too well,
Its hapless victim best can tell;
His wretched days, and sleepless nights—
His deep despair, and waking frights,

Create the wish that life were past,
And each returning sun the last;
Thus outraged nature vengeance takes,
When recklessly her laws he breaks.

---

### CANTO SECOND.

### THE ARGUMENT.

**Effects of the fetid and poisonous effluvia arising from decayed teeth upon the system, when inhaled by the lungs, and disseminated through the blood. Penalty attached to violated laws. The victim of toothache described. His soliloquy while enduring its torments. The tooth's reply, setting forth the certain consequences of neglect, and the superiority of natural over artificial teeth.**

Once more but give the teeth a prey
Successive victims to decay,
The lungs revolt at every breath,
Compelled to breathe the poisonous death
From carious portions, once so fair,
Infecting all the vital air.
By each electric filament,
This dread miasma wide is sent
  Throughout the suffering frame,—
Through quivering pulse, and throbbing heart,
Invading every vital part,
  As some devouring flame,
Wildly raging, flashing, burning,
Health and life to ruin turning.

This misfortune, though so great,
May be averted, 'tis not fate.
'Tis man himself who will not hear,
Or yet to reason lend an ear,
Who turns him deaf to nature's voice,
And makes forbidden things his choice;—
Then must the penalty be paid,
Though on the ruin he has made,
He oft may turn with anguished eye,
And heave the long, deep, hopeless sigh.
No laws of Heaven were made unneeded,
Once let them be by man unheeded,
In many a form of suffering, they
Return to judge, once cast away;
And one, more poignant than the rest
Of anguish, pain, and deep unrest,
Arises dark, and hated still,
The strength combined of every ill.
Look, while a picture we shall trace—
'Tis of a human form and face,—
That haggard look, that wretched mien,
And such strange grimaces, I ween,
Such frowns and scowls are seldom seen;
With pallid brow, and sunken eye,
And many a deep, despairing sigh,—
With toilet careless, uncombed hair,
All pointing upward here and there,

And reckless, wild, abstracted air-——
A maniac! is he? let's draw near,
His fatal malady to hear;—
In screams, in words, now quick, now slow,
He thus reveals his tale of woe:

O savage pang! O keenest torture!
Horrid! wild! Oh! murderous pain!
'Twill drive me quite to fury—madness,—
Distracting, tearing all my brain!

If once I sleep, there comes a phantom—
A monster—hateful—fiendish thing,
Creeping o'er me as the shadow
From a demon's cursed wing.

Sights and sounds appalling! hideous!
Gather round me grim and dread—
Noises like ten thousand thunders,
Crash and shiver through my head!

Lashed by snaky tongues, envenomed,
Stared by hellish, fiery eyes,—
Worse than haunted by the furies!
Earth and heaven shall hear my cries!

Gather up all forms of suffering
Known to the human frame—
Aye, blend them in one pang of torment—
*Detested toothache is the name!*

Yes, aching tooth, *thou* art the monster!
Deepest vengeance shalt thou know,—
*Thou* hideous fang! shalt feel the vulture
That tears my bursting head with woe!

My unstrung nerves shall hail the moment,
That bids thy maddening torments cease;
Shall spurn thee with unmeasured scorning,
And bring my writhing spirit peace.

He ceased to speak, but uttered shrill,
A mortal cry that haunts me still;
For the vexed tooth, to fury stung,
To hear such words from mortal's tongue,
With phrenzied throb, intenser yet,
Poured forth its wrongs, and its regret;
And these the truths which from it sped—
Hear what this tooth in anguish said:

" Denounce me, wrong me as thou wilt,
Whose is the wrong, and whose the guilt?
Hadst thou but treated me with care,
I had not known decay—
A constant friend through all thy life,
How long soe'er its day.

Few years ago, when dazzling, white,
I first came forth to greet the light;
When in this arch I took my place,
To render perfect thy young face.
I stood aghast, and trembling saw
How at defiance every law,
Designed thy dentals to protect,
Was set at naught by thy neglect.
How first came ices, freezing, chill,
That made each nerve with terror thrill!
Then acids keen, then liquids came,
Just boiling from the fiercest flame,
Thy teeth t' assail—need'st wonder thou
That they all lie in ruin now?
Frequent ablutions found us not,
And brush and dentifrice forgot,
By stealth insidious tartar came,
And seized upon our mortal frame;
Dread caries, too, ere long appears,
The plague-spot of a thousand fears.
There's one, whose counsel hadst thou sought,
Enduring life to me had brought,—
So nicely fashioned by his hand,
As 'twere a magian's mystic wand,
Had been the golden foil to thee
More valued than the brightest gem
Set in a monarch's diadem,
Or other priceless thing could be.
Its wonder-working spell of power
Had saved thee many a luckless hour
Of bitterest pain, almost despair,
Which thou hast borne, and still must bear;
Soon from my hated presence free,
The rest ere long will follow me,
And thou a toothless wight shalt be;
But in thy joy, do not forget
The one wild crash in keeping yet,
When that tremendous grasp shall come,
To tear me from my rightful home.
Though prompt successors may be thine,
By hand of art, as pure to shine,
But for the causes I have shown,
No such misfortune hadst thou known;
This agony had not been felt,
And unmolested we had dwelt,
Imparting ever to thy face
A touch of beauty and of grace.

### CANTO THIRD.

### THE ARGUMENT.

Perfection of the Creator's gifts, and their evident design to last as long as life is continued to us. Inevitable derangements, consequent upon indulgence in luxuries, felt by all the nerves of the system, and especially their effect upon the dental nerves, Entreaty to banish this source of so many woes. Allusion to the long life of Wordsworth, evidently the result of his simple habits. The objector who may think we have overrated dentistry, answered. The science and its practice annihilated. An example of affright and sorrow consequent upon this. Importance of perfect teeth to public speakers.

The gifts of God were perfect made,
And every flower and grassy blade
A purpose wise was meant to serve,—
Thus brain and muscle, tooth and nerve,
Instinct with life, should long remain
Strangers to disease and pain,
And each perform its perfect trust,
Until the parted spirit must
Yield up its clay to kindred dust.
Alas that man should ever be
Content to bow the willing knee,
An abject slave to luxury!
His life, his energies to waste,
To gratify a morbid taste,
Which fell disease ere long must bring,
Deadly as the viper's sting.
And every member needs must feel
The system's general woe or weal.
Each dental nerve but feels too well,
Indignant in its ivory cell,
    The discord raging on its way,
For aid most earnestly to call,
It beats against its prison wall,
    Fast crumbling to decay.
Then bid this wily monster flee
Alluring, artful luxury!
    And with sighs and tears,
Then man with health his heart may cheer—
Like rural bard of Windermere,
    Attain his fourscore years.
Or like the mightiest seer of old,
O'er whom centennial years had rolled
With stately step, unfurrowed cheek,
And aspect holy, pure, and meek,—
Unfailing voice, unfading eye——
What glory thus like him to die!

Mayhap there be who think, or dream
That we around our favorite theme
Have thrown importance in our song
Which never justly can belong
To this, they say, "which can but claim
Among mechanic arts a name."
But know ye not it must combine
With precious lore from learning's shrine,
The artist's taste, the surgeon's skill,
Its varied duties to fulfill?

Let *such* but listen for a space,
While we from every human face,
Annihilate entire
All dentistry has ever done,
And every triumph it has won—
Bid e'en its name expire.
Would not consternation then
Pervade entire all ranks of men?
As when of old the fair Iduna
Vanished from that dreamy land,
The dim old mansions of Valhalla
From Odin and his shadowy band?
With shrunken form and furrowed brow,
Aghast they stand! behold them now!
White-haired and toothless are they all,
And vainly on the goddess call.
No hand but hers can youth restore,
If she return to them no more,
Unceasingly they must deplore.

Thus were the dentist and his art
Once banished from the earth,
What woes would seize on many a heart,
And circle round each hearth!
Beside a home-hearth let us rest,
With health, and hope, and plenty blest!
The evening fire is burning bright,
And many a thrill of deep delight
Each silvery tone of music brings,
While Ida plays and sweetly sings;
Glad sounds discourse the ivory keys,
Beneath her touch of grace and ease—
And her betrothed is by her side,
And gazes on his destined bride
With joy intense, and manly pride;
He drinks the music of each tone,
And blesses Heaven that he alone
Is the sole object of her choice,
She of the sweet, enchanting voice;—

But look! what wonder seizes all!
Her words in broken lispings fall!
The song is hushed, and tears arise—
"My teeth! my teeth!" the fair one cries.——
Thy Chosen! Ida, Where is he?
Transfixed he stands, the change to see—
Those beauteous pearls! and can it be
The artist fashioned with such care,
And set them with device so rare?
Sure nature's self must well nigh yield,
And art, victorious, take the field.
But Ida's woes are not alone,
Now from the sofa comes a groan—
Her sire hath fallen on his cheek,
And little Ellen scarce can speak;
For toothache, once compelled to flee,
Returns with tenfold agony,

And pain and sorrow reign supreme,
Where all was blissful as a dream;
And this is but a single case,
Where tens of thousands we might trace.
The sacred desk, whose champions wise
Would ever point us to the skies;
The bar of justice, halls of state,
Where patriots swell the high debate,
In contest o'er a nation's fate,
Our luckless change, methinks would feel,
And many a noble form would steal
Away in silence from the place,
With faltering words and saddening face.

The voice, this agent of the mind,
In a material form enshrined;
This gift of speech, this thing of power,
Has wielded oft, in danger's hour,
A weapon for the truth and right,
Of loftier aim and surer might,
Than thousand practiced blows can deal
From surest hand and truest steel.
Though vocal chords and lungs were made,
This gift divine of speech to aid,
Without the dentals must it be
Devoid of strength and harmony.
Go, ask yon gifted child of song,
Who sways at will the enraptured throng,
If this sweet spell, this trancing power,
Could last but for a single hour,
Without these aids to that sweet voice,
Which bids the listener's soul rejoice.

---

### CANTO FOURTH.

### THE ARGUMENT.

What personal beauty, in many instances, owes to the dental art. Reply to one who would despise beauty, which was a divine gift, and pronounced good by its Author. Impossibility of any just claim to beauty, without bestowing strictest care upon the teeth. Invocation to youth. Address to mothers. Their ability to render their offspring what nature designed them to be, physically, intellectually, and morally. Watchfulness of the teeth enjoined as a means of securing a perfect physical organization. Importance of what may seem trifling. The loveliness of a sweet smile, and the charm of a perfect voice, neither of which can be perfect without fine teeth. Conclusion.

The wise, the useful, and the true,
Our thoughts have shared, and beauty, too.
Its claims unheard, must not give o'er,
While art can one great charm restore.
Rejoice! O thou! who hast the skill
To form and fashion at thy will
The beauty of the face divine;
And ye who bow at beauty's shrine,
Know ye how much the dental art
Has power to sway the human heart?
There is, within its inmost cell,
A deathless love of beauty's spell—

A love of that bewitching grace,
Seen oftenest in a lovely face.
And manly beauty in its pride,
(As well as the gentler one beside),
With the lofty brow and flashing eye—
The stately form, the purpose high,
O'er the features proudly beaming,
Almost godlike in their seeming.
Magician of charms, oft flees to thee,
Restorer of grace and harmony!
But one, who would be over-wise,
"Beauty! amazed," most gravely cries
"What is it worth? it fades away,
The flowret of a summer day."
But was it not in wisdom given—
A precious thing, a gift of Heaven?

When sky, and stars, and beaming sun,
And wide creation's work was done;
When fragrant blossoms pendant hung,
And every bough with music rung,
And sparkling wings arose in air,
And all was passing sweet and fair;
Then by th' all-seeing One, the Just,
Was fashioned from the sleeping dust,
A being great, and pure, and high,
With look of Heaven in his eye;—
When still more beautiful there came
His like in form, yet not the same.
When perfect, lovely woman stood,
The Sov'reign said: "*All this is good*"—
"'Tis *very* good!" hear Heaven declare;
And do not heavenly natures wear
Celestial beauty still more fair?
Then is it not a blessed boon?
Though when neglected, fading soon;
For want of care in life's young day,
The fairest charms soon fade away.

'Twere vain for sparkling eye to beam
O'er features lovely as a dream,
With changing cheek of roseate glow,
And sunny curls, and neck of snow;
Oh! fair one, hear thy certain doom,
In which thy charms must find their tomb;
Vain, all vain, this sweet array,
If once the parted lips display
Half ruined teeth, of ebon shade,
Once gems of pearl, so perfect made;
'Twas sad neglect this ruin wrought,
With loss of fairest beauty fraught.
The Dentist seek—alone his skill
  Can e'er thy hapless loss restore,
With ready hand and generous will,
  Or beauty's reign is thine no more;
And let these charms external be
The mirror of that purity

Which dwells within the exalted soul—
In harmony, a perfect whole.

All hail to thee, Oh! joyous youth!
Sweet hours of innocence and truth!
How rapturous are thy deep delights!
Thy sunny days, thy tranquil nights!
Glad as the bird's sweet voice in spring,
That sips, while poised on shining wing,
The nectar of a thousand flowers,
So gladly speed our youthful hours;
Oh! why so quickly haste they on?
A smile—a breath—and they are gone!
There's *one* who hath a wondrous skill,
In youth to make us what she will;—
To *thee*, O, mother, do we fly,
With throbbing heart and anxious eye,
To thee we come because we must—
Thine is the power, and thine the trust.
'Tis *thou* canst form each infant mind
To be what wisely, Heaven designed;
Thine influence is its beacon ray—
Oh! let it be a star of day!
And not the meteor's transient glow,
Not borean lights o'er waste of snow,
Beaming while all is chill below.
'Tis thus with the material frame,
As with the mind it is the same.

What mother's heart would not rejoice,
And raise a grateful eye and voice,
Upon her darling's face to see,
Instead of pale deformity,
A Hebe glow of rosy health,
That purest treasure, priceless wealth;
Then let her seek the cause to know
From which derangements frequent flow,
Nor think a trifle needs no skill—
Search out each latent cause of ill;
And prominent among them all,
The teeth for strict attention call;
Many a victim of dentition
Hath found an early grave,
Because the mother's fearful heart
Refused to trust the hand of art—
The only power to save.
When once these days of peril o'er,
And banished fears return no more,
The *second* teeth as much may need
A watchful care, as constant heed;
Again the artist's hand may aid
What nature has imperfect made;
Irregular let them remain,
Future regrets will all be vain,—
Well may they be, in after years,
The source of sorrow and of tears.

"'Tis but a *trifle*," one may say—
"An unimportant thing"——
Know ye that trifles round our way
Unnumbered ills may bring;

Or they may weave with woof of light,
All rainbow-hued, and dazzling bright,
The threads of destiny;
The minute elements of beauty
Must each one perfect be—
Once break the magic circle,
Quick dies the harmony.

Oh! there's a world of beautiful things
That wander in air, on glorious wings,
And fairy-like sounds that glide along,
Sweet as the tones of a minstrel's song;
We love them well as they speed us by,
These beautiful forms of earth and sky!
There are things *more* charming, aye, dearer still,
And they sweep our hearts with a joyous thrill;
No lovely sound, though music's own,
Is so sweet as the voice of perfect tone;
Its gentle notes, so glad and clear,
Are ever a joy to the listener's ear;
No thing of beauty or form of grace
So sweet as the smile from a loved one's face—
The voice and the smile—they have high control
To cheer the heart and to sway the soul.

But the voice must be perfect, the smile be as sweet,
Or to give this high pleasure they can not be meet;
Though blushes may glow on the fair young cheek,
And wondrous things the eye may speak,
If the lips, in parting, disclose to view
The teeth imperfect, or dusk in their hue,
The spell of the smile shall vanish in air,
Though all other charms be graceful and fair:
All glorious things may pass on, the while,
But leave us the light of a beautiful smile,
And the music leave of a dear one's voice,
Whose trancing tones bid us ever rejoice—
The voice and the smile—they have high control
To cheer the heart, and to sway the soul.

Strong words, methinks, can ne'er reveal
The gratitude which thousands feel
To those who life's best years have lent,
And thought and energy have spent,
Dental science to impart,
And banish with consummate art,
As by magician's wondrous powers,
The thousand ills which once were ours.
Their genius true has made them known
Far as the light of truth has flown;—
There's room for Parmlee's—Brewster's name
A niche within the halls of fame.

*Others* we might mention, too,
*Devoted*, *courteous*, *and as true*—
Aye, them to whom this simple song,
Though all unworthy, must belong.
What though their name to them descended,
With high ancestral honors blended—
What though the gorgeous palace rose
For sires of theirs, who found repose
In those princely halls, where the exiled great,
In after years, brought a monarch's state;
And India laid her golden trust
At another's feet, now sleeping in dust;
And Moultrie might tell of contest won,
Of dying sire and dauntless son—
Of patriot sire, in battle strife,
Calmly yielding up his life,
Bequeathing his son his blood-dyed sword,
With a hero's soul and a conqueror's word;
Yet they trust them not to deeds gone by—
For honors past disdain to sigh;
And I, mayhap, incur their blame,
That I've *presumed* these things to name.
Whatever honors they have gained,
Or high success may have attained,
They owe to effort and to skill—
To a truthful heart and a generous will.
God speed ye on your destined way
With each returning day,
With blessings high, whate'er betide,
And the brightest star of Heaven to guide.

